# Influencing factors and model predictive analysis of the efficacy of adjuvant ¹³¹I therapy after DTC surgery

**DOI:** 10.3389/fonc.2026.1887096

**Published:** 2026-07-10

**Authors:** Honghong Pan, Mingming Zheng, Qian Su

**Affiliations:** 1 Tianjin Medical University Cancer Institute and Hospital, National Clinical Research Center for Cancer, Tianjin’s Clinical Research Center for Cancer, Tianjin, China; 2 Tianjin Cancer Hospital Airport Hospital, National Clinical Research Center for Malignant Tumors, Tianjin, China

**Keywords:** adjuvant radioiodine-^13^1 therapy, differentiated thyroid carcinoma (DTC), prediction model, prognosis, risk factors

## Abstract

**Objective:**

To explore influencing factors and construct predictive models for the efficacy of postoperative adjuvant ¹³¹I therapy in patients with differentiated thyroid cancer (DTC).

**Methods:**

A total of 376 DTC patients who underwent total thyroidectomy and received initial adjuvant ¹³¹I therapy at a fixed activity of 100 mCi were retrospectively enrolled. All patients were divided into excellent response (ER) and non-excellent response (nER) groups according to the 2025 ATA evaluation criteria at the 6-month follow-up. Relevant statistical methods and multivariate binary logistic regression were adopted to screen independent influencing factors. ROC curve analysis, support vector machine (SVM) and random forest (RF) were applied to establish predictive models.

**Results:**

The incidence of nER was 48.40%. BMI, number of metastatic lymph nodes (LNM), stimulated thyroglobulin (s-Tg), BRAF mutation (BRAF) and mean residual ¹³¹I uptake count (C-mean) were independent risk factors (OR = 1.112, 1.061, 1.198, 3.041, 1.103, respectively), while maximum residual ¹³¹I uptake count (C-max) was an independent protective factor (OR = 0.995). The AUC of logistic regression model was 86.30%, and optimal cut-off values were determined as follows: BMI 29.345 kg/m², LNM 9.5 nodes, s-Tg 3.3 ng/ml, C-max 18.5 counts, and C-mean 38 counts. The AUC of SVM model was 86.33%, and the RF model showed the optimal predictive performance with an AUC of 90.77%. S-Tg, LNM and BMI were the top three important predictors. Patients with concurrent high s-Tg, multiple metastatic lymph nodes and elevated BMI had a 100% risk of nER.

**Conclusion:**

A considerable proportion of DTC patients fail to achieve ER after 100 mCi adjuvant ¹³¹I therapy. BMI, LNM, s-Tg, BRAF and C-mean are independent risk factors for nER, while C-max are independent protective factors against nER. The RF model possesses excellent predictive ability for adjuvant ¹³¹I treatment response. The coexistence of s-Tg ≥ 3.3 ng/ml, LNM ≥ 9.5 nodes, and BMI ≥ 29.345 kg/m² indicates an extremely high risk of nER, which offers evidence-based support for the judicious escalation of ¹³¹I activity in selected patients undergoing adjuvant therapy.

## Introduction

Adjuvant therapy, in addition to ablating thyroid remnants, aims to eradicate suspicious occult residual or metastatic lesions that cannot be confirmed by postoperative imaging, so as to improve disease-specific survival and progression-free survival and reduce the tumor recurrence rate. The 2025 ATA guidelines specify that for initial adjuvant ¹³¹I therapy [for suspected microscopic residual disease in differentiated thyroid cancer (DTC) without distant metastases], the recommended administered activity is generally higher than that used for remnant ablation and up to 150 mCi, with the specific activity determined by postoperative risk stratification. Controversy exists regarding whether a higher activity can more effectively reduce the recurrence of DTC compared with a lower one (1–4). In previous studies, many scholars have put forward guiding recommendations for adjuvant therapy based on retrospective analyses. However, due to insufficiently strict control of the definition of “adjuvant therapy”, these studies are susceptible to selection bias. Based on clinical guidelines and previous studies, this study defines “adjuvant ¹³¹I therapy” as the initial administration of ¹³¹I therapy in DTC patients after total thyroidectomy—who are stratified as intermediate-high or high recurrence risk, and/or have high stimulated thyroglobulin (s-Tg) levels (s-Tg > 10 ng/mL)—in whom no definite residual or metastatic lesions were detected by conventional imaging (including cervical ultrasound, neck and chest CT, and whole-body bone scintigraphy). On the basis of clarifying the definition of adjuvant ¹³¹I therapy, this study retrospectively analyzed the independent influencing factors for the efficacy of adjuvant ¹³¹I therapy and constructed predictive models, aiming to provide more reliable evidence and more accurate guidance for adjuvant ¹³¹I therapy in patients with DTC after surgery.

## Materials and methods

### Study population

A retrospective study was conducted on 376 patients with DTC who underwent total thyroidectomy and received initial adjuvant ¹³¹I therapy at Tianjin Medical University Cancer Institute and Hospital, with a uniform therapeutic activity of 100 mCi for all patients. Among them, 170 were male and 206 were female, aged 13–74 years, with a median age of 40.50 (interquartile range [IQR], 33.25–53.00) years. Based on conclusions from relevant authoritative studies, we recorded relevant indicators that may affect the efficacy of adjuvant therapy, including gender, age, body mass index (BMI), maximum diameter of primary cancer lesions (MD-CL), number of cancer lesions (CL), maximum diameter of lymph node metastatic lesions (MD-LNM), number of lymph node metastases (LNM), with or without lymph node capsular invasion (LNCI), time interval from total thyroidectomy to ¹³¹I therapy (Ptime, postoperative interval), s-Tg, thyroid-stimulating hormone (TSH), with or without BRAF mutation (BRAF), with or without Hashimoto’s thyroiditis background (HB), maximum count of residual uptake foci on thyroid imaging (T-max), mean count of residual uptake foci on thyroid imaging (T-mean), maximum count of residual uptake foci on ¹³¹I imaging (C-max), and mean count of residual uptake foci on ¹³¹I imaging (C-mean).

### Inclusion criteria

1. Pathologically confirmed DTC after total thyroidectomy;
2. No prior radiotherapy, chemotherapy, or other anticancer treatments administered postoperatively;
3. No distant metastases at baseline;
4. Stratified as having intermediate-high or high recurrence risk, and/or with high s-Tg levels (s-Tg > 10 ng/mL), with no definite residual or metastatic lesions detected by conventional imaging (including cervical ultrasound, neck and chest CT, and whole-body bone scintigraphy);
5. Consistent detection methods for the same indicator and consistent examination protocols for the same imaging modality across all patients;
6. Discontinued levothyroxine sodium tablets (or other thyroid hormone preparations) for 2–4 weeks and adhered to a low-iodine diet prior to ¹³¹I therapy;
7. Complete demographic and clinical data available for all patients.

### Exclusion criteria

1. Did not meet the definition of “adjuvant ¹³¹I therapy” as defined in this study;
2. Positive thyroglobulin antibody (TgAb) status (to ensure reliable measurement of s-Tg);
3. Definite metastatic lesions detected on post-therapy ¹³¹I whole-body scan (Rx-WBS);
4. Severe dysfunction of vital organs;
5. Severe comorbidities;
6. Concomitant other malignancies;
7. Incomplete clinical and laboratory data.

### Grouping criteria

According to s-Tg measurements and imaging findings at 6 months of follow-up after adjuvant ¹³¹I therapy, patients were divided into the excellent response (ER) group (n=194) and the non-excellent response (nER) group (n=182) per the response evaluation criteria specified in the 2025 ATA guidelines (3, 4). The nER group accounted for 48.40% of all patients.

### Parameter measurement methods

S-tg and TSH were tested with consistent dedicated assays for all patients. Thyroid scintigraphy and whole-body ¹³¹I scintigraphy were acquired with the DR 870 SPECT system, and identical acquisition parameters and scanning durations were set for the same imaging protocol in all patients. Whole-body ¹³¹I scans were performed on day 3 after radioiodine administration. All semi-quantitative analyses were completed on the Xeleris post-processing workstation. Regions of interest (ROIs) were manually delineated on anterior planar images to derive four metrics: T-max (maximum count value of residual uptake lesions on thyroid scintigraphy), T-mean (mean count value of residual uptake lesions on thyroid scintigraphy), C-max (maximum count value of residual uptake lesions on ¹³¹I scintigraphy), and C-mean (mean count value of residual uptake lesions on ¹³¹I scintigraphy). All ROIs were independently delineated by nuclear medicine physicians with at least three years of working experience, and the mean values of the two sets of measurements were adopted for subsequent analyses.

### Statistical analysis

The Shapiro-Wilk test was used for normality analysis of continuous data. Non-normally distributed continuous data were expressed as median (interquartile range [IQR], P25–P75), and categorical data were expressed as numbers (n) and percentages (%). The Mann-Whitney U test, chi-square test, and multivariate binary logistic regression analysis were used to identify the independent influencing factors for nER after adjuvant ¹³¹I therapy. ROC curve analysis, support vector machine (SVM), and random forest (RF) algorithms were applied to construct predictive models, so as to analyze and determine the optimal cut-off values of indicators and the feature importance of the models.

**Figure 1:**
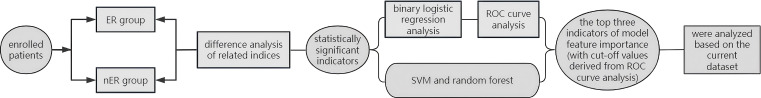
Flowchart of the paper analysis.

The analysis process of the entire paper is shown in **Figure 1**:

## Results

Univariate comparisons revealed statistically significant differences between the two groups in age, BMI, MD-LNM, LNM, s-Tg, BRAF, HB, T-max, T-mean, C-max, and C-mean (all *P* < 0.05; **Table 1**).

**Table 1:** Univariate analysis of related indicators between the two groups.

Variables			ER group	nER group	z/*χ^2^*	*P*
**N**			**194**	**182**	**-**	**-**
Sex		Male(n,%)	80(41.24%)	90(49.45%)	2.557	0.110
		Female(n,%)	114(58.76%)	92(50.55%)		
Age(year old) M(*P*_25_,*P*_75_)			43(34,54)	39(33,51)	-2.733	0.006
BMI(kg/m^2^) M(*P*_25_,*P*_75_)			24.97(22.08,27.52)	26.99(23.37,31.25)	-4.529	<0.001
MD-CL(cm) M(*P*_25_,*P*_75_)			1.3(1.0,2.0)	1.5(1.0,2.0)	-1.459	0.145
CL(n) M(*P*_25_,*P*_75_)			3(2,4)	3(2,6)	-1.335	0.182
MD-LNM(cm) M(*P*_25_,*P*_75_)			0.9(0.5,1.3)	1.0(0.6,1.6)	-2.056	0.040
LNM(n) M(*P*_25_,*P*_75_)			8(5,12)	11(8,18)	-6.743	<0.001
LNCI (n,%)			157(80.93%)	150(82.42%)	0.139	0.709
Ptime(months) M(*P*_25_,*P*_75_)			2(1,2)	2(1.75,3)	-1.301	0.193
s-Tg(ng/ml) M(*P*_25_,*P*_75_)			1.58(0.355,4.673)	7.505(4.56,13.6)	-11.080	<0.001
TSH(mIU/L)	>30,≤60(n,%)		19(9.79%)	26(14.29%)	-4.88	0.625
	>60,≤100(n,%)		66(34.02%)	55(30.22%)		
	>100(n,%)		109(56.19%)	101(55.49%)		
BRAF (n,%)			157(80.93%)	169(92.86%)	11.591	0.001
HB(n,%)			61(31.44%)	25(13.74%)	16.690	<0.001
T-max(counts) M(*P*_25_,*P*_75_)			1(1,41.25)	22.5(1,46.5)	-2.493	0.013
T-mean(counts) M(*P*_25_,*P*_75_)			1(1,27)	8.5(1,31)	-2.321	0.020
C-max(counts) M(*P*_25_,*P*_75_)			54(15,134)	65.5(23,156)	-2.223	0.026
C-mean(counts) M(*P*_25_,*P*_75_)			15(3,33.25)	20(7,51)	-2.531	0.011

Indicators with statistically significant differences (age, BMI, MD-LNM, LNM, s-Tg, BRAF, HB, T-max, T-mean, C-max, C-mean) were included in the multivariate binary logistic regression analysis. The results showed that BMI, LNM, s-Tg, BRAF and C-mean were independent risk factors for nER after adjuvant ^131^I therapy, while C-max were independent protective factors against nER, as shown in **Table 2**.

**Table 2:** Multivariate binary logistic regression analysis of the efficacy of adjuvant ¹³¹I therapy.

Indicators	$\hat{\text{β}}$	*SE*	*Waldχ^2^*	*P*	*OR*	*95 %CI*
Age	-0.019	0.011	2.833	0.092	0.981	0.960-1.003
BMI	0.106	0.030	12.625	<0.001	1.112	1.049-1.179
MD-LNM	-0.021	0.146	0.020	0.887	0.979	0.736-1.304
LNM	0.059	0.020	9.153	0.002	1.061	1.021-1.103
s-Tg	0.181	0.029	37.816	<0.001	1.198	1.131-1.269
BRAF	1.112	0.454	5.997	0.014	3.041	1.249-7.406
HB	0.422	0.326	1.669	0.196	1.524	0.804-2.890
T-mean	-0.014	0.029	0.251	0.616	0.986	0.931-1.043
T-mean	0.040	0.045	0.798	0.372	1.041	0.953-1.136
C-max	-0.005	0.002	6.418	0.011	0.995	0.992-0.999
C-mean	0.013	0.006	4.860	0.027	1.013	1.001-1.025

**Figure 2:**
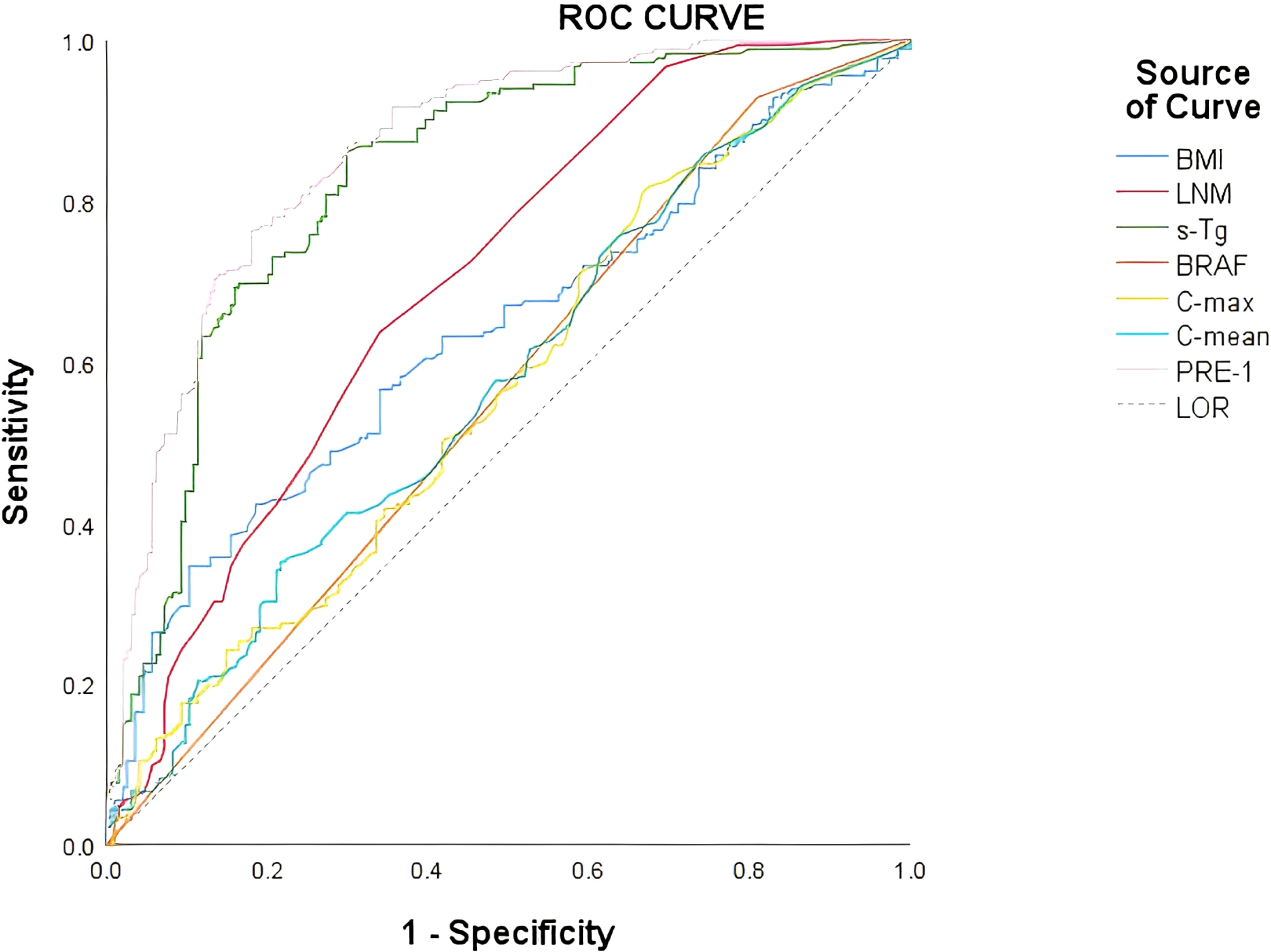
ROC curve analysis of BMI, LNM, s-Tg, BRAF, C-max, C-mean and PRE_1 on the outcome of adjuvant ¹³¹I therapy.

A new binary logistic regression model was constructed using the independent influencing factors (BMI, LNM, s-Tg, BRAF, C-max, and C-mean), and the predicted probability (PRE_1) was obtained for subsequent ROC curve analysis. The results showed that the binary logistic regression model exhibited good overall predictive performance for the efficacy of adjuvant ¹³¹I therapy, with an AUC of 86.30%, a sensitivity of 76.40%, and a specificity of 81.40%. The optimal cut−off values for the indicators were determined as follows: BMI 29.345 kg/m², LNM 9.5 nodes, s-Tg 3.3 ng/mL, C-max 18.5 counts, and C-mean 38 counts, as shown in **Table 3** and **Figure 2**.

**Table 3:** ROC curve analysis of the efficacy of adjuvant ¹³¹I therapy.

Indicators	AUC (*95% CI*)	Cut-off value	Sensitivity	Specificity	Youden index
BMI	0.635 (0.579-0.691)	29.345	0.346	0.897	0.243
LNM	0.701 (0.649-0.753)	9.5	0.637	0.660	0297
s-Tg	0.830 (0.789-0.872)	3.3	0.863	0.701	0.564
BRAF	0.560 (0.502-0.618)	–	0.929	0.191	0.12
C-max	0.566 (0.509-0.624)	18.5	0.819	0.325	0.144
C-mean	0.575 (0.518-0.633)	38	0.352	0.784	0.136
PRE_1	0.863 (0.826-0.900)	–	0.764	0.814	0.578

**Figure 3:**
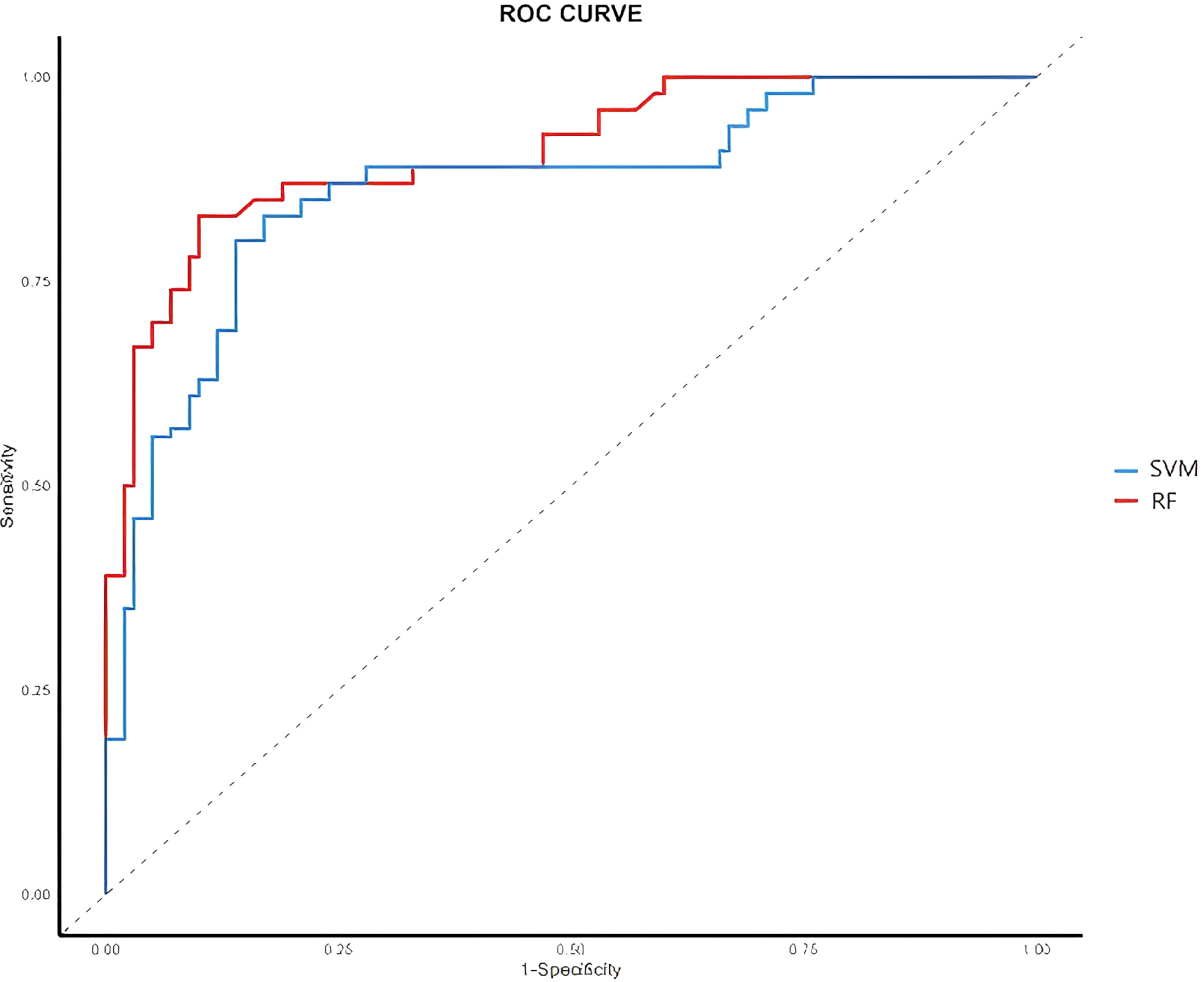
ROC curves of two prediction models for adjuvant ¹³¹I therapy efficacy: SVM, and RF.

Since the AUC of the binary logistic regression model from ROC curve analysis did not reach 0.90 and the sensitivity was below 0.80, two machine learning approaches (SVM and RF) were further used to construct predictive models for the efficacy of adjuvant ¹³¹I therapy. Model performance was evaluated using ROC curve analysis. The results demonstrated that the SVM model had an AUC of 86.33%, an accuracy of 80.34%, a sensitivity of 86.21%, and a specificity of 79.63%; the random forest model showed the best performance, with an AUC of 90.77%, an accuracy of 85.71%, a sensitivity of 87.93%, and a specificity of 83.33% (**Figure 3**).

**Figure 4:**
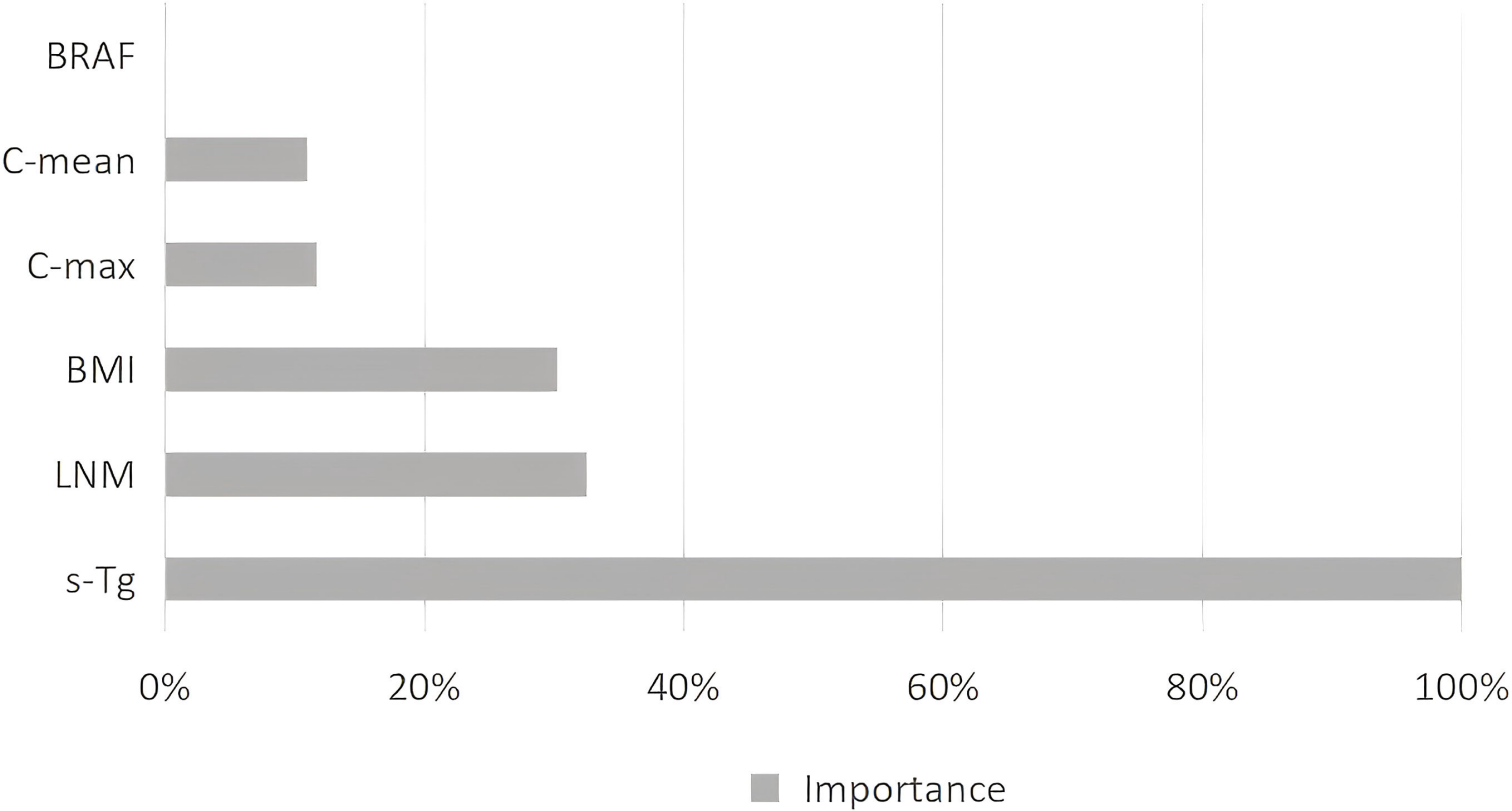
Feature importance ranking of the random forest model.

For RF model construction, Z-score standardization was first applied to numerical features to eliminate dimensional differences, and the categorical variable BRAF was converted to a factor type with explicit category labels. Stratified random sampling was used to split the dataset into a training set and a test set at a 7:3 ratio to ensure a consistent distribution of the target variable (ER/nER) across both subsets. A fixed random seed was set to guarantee the reproducibility of results. The model was trained using a repeated 10-fold cross-validation strategy to evaluate performance stability, with five repetitions specified to reduce random errors. The number of randomly selected features for each tree (mtry) was identified as the core hyperparameter for optimization, and a tuning grid with three candidate values (2, 5, 8) was constructed. Grid search was performed to select the optimal parameter combination, and the number of decision trees was set to 300 to balance model fitting ability and computational efficiency. The final model demonstrated favorable performance; s-Tg, LNM, and BMI ranked among the top three features in terms of importance, with s-Tg exhibiting the highest importance (**Figure 4**).

**Figure 5:**
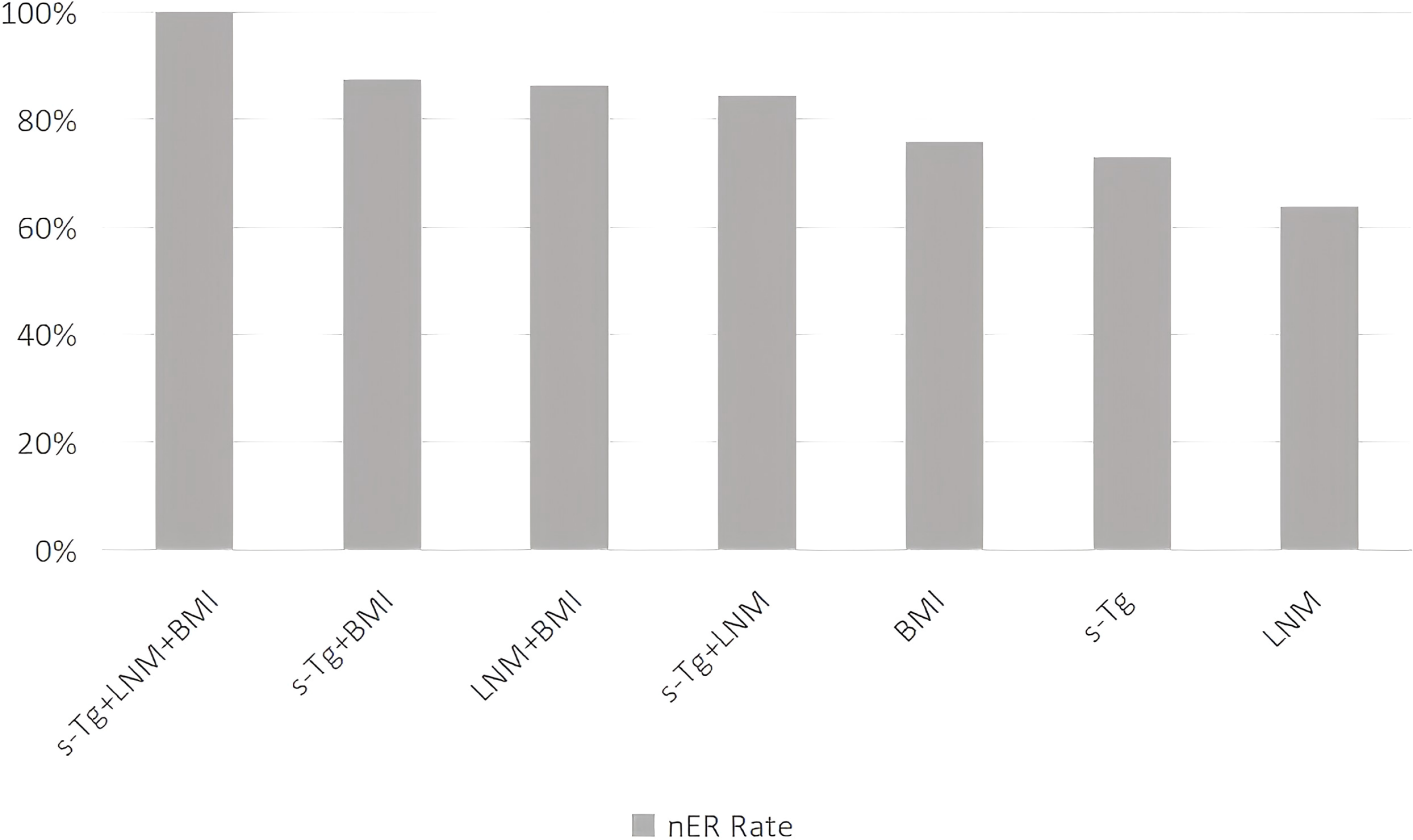
Percentage of patients with nER according to different risk factor combinations.

Based on the optimal cut−off values from ROC curve analysis and feature importance in the random forest model, the three indicators (s-Tg, LNM, and BMI) were included for further analysis. The results showed that the nER rate was 73.02% when s−Tg ≥ 3.3 ng/mL, 63.74% when LNM ≥ 9.5 nodes, and 75.90% when BMI ≥ 29.345 kg/m². The nER rate was 84.43% when s−Tg ≥ 3.3 ng/mL and LNM ≥ 9.5 nodes, 87.50% when s−Tg ≥ 3.3 ng/mL and BMI ≥ 29.345 kg/m², and 86.30% when LNM ≥ 9.5 nodes and BMI ≥ 29.345 kg/m². Notably, the nER rate reached 100% when s−Tg ≥ 3.3 ng/mL, LNM ≥ 9.5 nodes, and BMI ≥ 29.345 kg/m² were present simultaneously (**Figure 5**).

## Discussion

The incidence of thyroid cancer is increasing worldwide, and the vast majority (>95%) of DTC retain iodine uptake capacity, making them suitable for postoperative ¹³¹I therapy. ¹³¹I therapy plays an equally important role as surgical treatment and thyroid hormone suppression therapy. According to current research progress at home and abroad, it is advocated to administer ¹³¹I therapy selectively according to the patient’s condition. Patients stratified as intermediate-high or high risk of recurrence benefit more from ¹³¹I therapy than those at low or low-intermediate risk (5). ¹³¹I therapy is divided into three categories according to purpose: thyroid remnant ablation, adjuvant therapy, and radioiodine therapy for metastatic disease. Among them, adjuvant therapy is the most controversial because it targets suspicious microcancerous lesions rather than structurally proven lesions or distant metastases confirmed by imaging. Due to the subjectivity of the evaluation criteria for adjuvant therapy and large differences in the baseline characteristics of patients, the administered activity of adjuvant therapy is not standardized. Within the activity range of 100–150 mCi, controversy remains regarding whether a higher activity can reduce DTC recurrence more effectively than a lower activity (3).

The 2025 ATA guidelines point out that routine thyroid remnant ablation and adjuvant therapy should be considered for high-risk patients, and adjuvant therapy may be considered for intermediate-high risk patients (4). Studies have shown that although no clear optimal s-Tg cut-off value has been established for ¹³¹I adjuvant therapy decision-making, s-Tg > 10 ng/mL can be used as an indication for adjuvant therapy (5). In addition, the most common metastatic sites of DTC are regional lymph node metastasis, lung metastasis, and bone metastasis, with other sites being rare (6). Therefore, this study defines adjuvant therapy as initial ¹³¹I therapy administered to DTC patients after total thyroidectomy who are stratified as intermediate-high or high recurrence risk, and/or have high s-Tg levels (s-Tg > 10 ng/mL), with no definite residual or metastatic lesions detected on conventional imaging (including cervical ultrasound, neck and chest CT, and whole-body bone scintigraphy).

In this study, we exclusively enrolled patients receiving adjuvant radioiodine therapy (100 mCi) and excluded those who underwent remnant ablation alone, as the two patient groups exhibit substantial differences in clinical indications, baseline risk stratification, and therapeutic goals. Pooling these two cohorts would introduce marked heterogeneity and confound the identification of predictors specifically associated with adjuvant treatment outcomes. Accordingly, we limited our cohort to patients treated in the adjuvant setting to maintain a homogeneous study population.

We retrospectively enrolled patients who met the definition of “adjuvant therapy” in this study, all of whom received adjuvant ¹³¹I therapy at an activity of 100 mCi. Therapeutic efficacy was evaluated according to the 2025 ATA response evaluation system at 6 months post-treatment. The results showed that the proportion of patients with nER reached 48.40%, indicating that a considerable proportion of patients fail to achieve excellent response under 100 mCi adjuvant ¹³¹I therapy, and there exist risk factors for unsatisfactory therapeutic effect.

Many studies have reported that obesity is a related factor for tumor invasiveness or poor prognosis. A large-scale Korean study reported that a higher BMI is associated with tumor diameter > 1 cm, more advanced TNM stage, and minimal extrathyroidal extension (7). However, a cohort study of Caucasian populations found no association between BMI and invasiveness at DTC diagnosis or during follow-up after initial ¹³¹I therapy (8). Some studies have shown that BMI may have an additive effect on the invasiveness of papillary thyroid cancer but has no effect on the therapeutic response after high-activity ¹³¹I therapy (9). Another study reported that in a decision tree model excluding ¹³¹I therapy-related variables, the predictive importance of BMI reached 7.7%, and 3.8% in the model including all variables, indicating that BMI is one of the key factors affecting the prediction of persistent disease and recurrence (10). This study shows that BMI is an independent risk factor for nER after adjuvant ¹³¹I therapy and ranks third in the feature importance of the random forest model. In the present study, the nER rate was 75.90% when BMI ≥ 29.345 kg/m². This result may be related to the fact that most of the populations included in this study are at intermediate-high risk, and the negative impact of obesity is greater at a higher risk stratification. BMI in our study was measured after thyroid hormone withdrawal. Although this withdrawal protocol was uniformly applied to all patients, the potential influence of withdrawal-induced fluid retention and metabolic changes on BMI cannot be entirely excluded.

The number of LNM is a recognized indicator for evaluating recurrence risk stratification and guiding postoperative ¹³¹I therapy for thyroid cancer. Studies have indicated that more than 5 LNM is one of the most important factors associated with unsatisfactory efficacy of ¹³¹I therapy (11). A classification tree model analysis also showed that the number of LNM and the LNM ratio can predict the initial response to ¹³¹I therapy, with cut-off values of 5 and 30%, respectively (12). The cut-off value of the number of LNM for predicting the therapeutic response of ¹³¹I therapy in this study is 9.5 (the clinically applicable cut-off was set at ≥ 10), which is higher than that in most studies. This may be due to the fact that the ¹³¹I therapy activity in this study was uniformly 100 mCi for adjuvant therapy, excluding the conventional 30–100 mCi used for thyroid remnant ablation. Therefore, only a greater number of lymph node metastases would result in nER under such a higher therapeutic activity.

Tg plays a crucial role in guiding ¹³¹I therapy and evaluating therapeutic response. At present, an s-Tg level of 10 ng/mL is a widely recognized cut-off value and can also be used as a criterion for adjuvant therapy. Studies have shown that s-Tg < 2.35 ng/mL can be used as the optimal cut-off value to predict that patients achieve disease-free survival after treatment (13). Wen et al. reported that the cut-off values of s-Tg for predicting ER in intermediate- and high-risk DTC patients after ¹³¹I therapy were 3.52 ng/mL and 1.04 ng/mL, respectively (14). Another study evaluating 296 intermediate- and high-risk DTC patients undergoing ¹³¹I therapy found that the risk of disease recurrence was significantly higher when the s-Tg level was above 5.21 ng/mL (15). In this study, s-Tg was the most important indicator for predicting the efficacy of ¹³¹I therapy, with a cut-off value of 3.3 ng/mL, which is consistent with the above studies. It can be considered that adjuvant ¹³¹I therapy is required when s-Tg > 10 ng/mL, while 100 mCi adjuvant ¹³¹I therapy is likely to fail to achieve ER when s-Tg > 3.3 ng/mL, which also explains why the ATA guidelines recommend an activity of 100–150 mCi for adjuvant ¹³¹I therapy. Although current studies have not proven that a higher activity is more effective than a lower activity, it can be seen from this study that 100 mCi adjuvant ¹³¹I therapy is not sufficient to achieve a satisfactory ER rate.

In DTC, the prevalence of BRAF mutations reaches 50%-80%, and BRAF mutation acts as a driving factor in the development and progression of thyroid cancer. The 2025 ATA guidelines indicate that isolated BRAF mutations represent an intermediate-risk molecular subtype in DTC. Compared to BRAF wild-type patients, patients with isolated mutations exhibit diminished tumor radioiodine uptake capacity, lower Tg seroconversion rates, higher recurrence risks, and relatively limited efficacy of radioactive iodine therapy. Furthermore, when BRAF occurs with concurrent mutations such as TERT, the correlation with increased recurrence risk and tumor-specific mortality becomes more prominent (4). However, a comprehensive analysis incorporating 14 studies demonstrated that BRAF mutations are significantly associated with impaired radioiodine uptake capacity in PTC patients but show no significant association with clinical response to iodine therapy or recurrence rates (16). In this study, BRAF mutations were identified as an independent risk factor for reduced efficacy of iodine-131 adjuvant therapy, indicating that BRAF mutations weaken the therapeutic effect of iodine-131.

Residual uptake on ¹³¹I imaging after ¹³¹I therapy can directly reflect the status of thyroid remnants. Studies have shown that at a median follow-up of 6.75 months after ¹³¹I therapy, the peak and mean absolute radioactivity concentrations (kBq/mL) in the group with disappearance of uptake lesions were significantly higher than those in the group with persistent uptake, while there was no statistically significant difference in the peak and mean standardized uptake values (SUV) between the two groups (17). In this study, C-mean was a risk factor for nER, while C-max was a protective factor against nER. The different prognostic effects of C-mean and C-max may be due to the fact that a high mean count indicates high total ¹³¹I uptake, reflecting more thyroid remnants or occult lesions, which may be difficult to eradicate completely with a single course of ¹³¹I therapy. In contrast, a high maximum count indicates high local ¹³¹I uptake in thyroid remnants or occult lesions, and the high-energy radiation exerts a strong ablative effect, which is more likely to achieve an excellent therapeutic effect. The relationship between the overall or local ¹³¹I uptake of lesions and therapeutic effect is complex, and more accurate analysis requires further comparison with the size and volume of thyroid remnants.

The random forest model constructed in this study shows the best performance, with an AUC of 90.77%, an accuracy of 85.71%, and a sensitivity of 87.93%, indicating favorable model performance. s-Tg, LNM, and BMI rank the top three in terms of feature importance. Based on the optimal cut-off values of the indicators and the feature importance of the model, regression analysis of the data in this study found that the nER rate reached 100% when s-Tg ≥ 3.3 ng/mL, number of lymph node metastases ≥ 9.5 nodes, and BMI ≥ 29.345 kg/m² were satisfied simultaneously. It is evident that when the aforementioned conditions are met, a 100 mCi adjuvant therapy dose is unlikely to achieve a satisfactory ER rate, and a higher dose of iodine-131 therapy may be employed instead.

## Data Availability

The raw data supporting the conclusions of this article will be made available by the authors, without undue reservation.
